# Re-exposure to endotoxin induces differential cytokine gene expression in the rat hypothalamus and spleen

**DOI:** 10.1016/j.bbi.2009.02.009

**Published:** 2009-08

**Authors:** Adriana del Rey, Anke Randolf, Johannes Wildmann, Hugo O. Besedovsky, David S. Jessop

**Affiliations:** a Dept. Immunophysiology, Institute of Physiology and Pathophysiology, Medical Faculty, Philipps University of Marburg, 35037 Marburg, Germany; b Henry Wellcome Laboratories for Integrative Neuroscience and Endocrinology (LINE), University of Bristol, Dorothy Hodgkin Building, Whitson Street, Bristol BS1 3NY, United Kingdom

**Keywords:** Lipopolysaccharide, Corticosterone, Stress, Cytokines, Hypothalamus, Spleen, Catecholamines, Endotoxin

## Abstract

This study was designed to investigate whether the pattern of hypothalamic and splenic cytokine expression induced by peripheral administration of a bacterial lipopolysaccharide (LPS) is affected by prior exposure to LPS derived from another bacterial strain. Injection of LPS from *Salmonella enteritidis* (LPS_2_) alone resulted in increased hypothalamic gene expression of IL-1β, IL-6, TNFα, IL-1ra and IL-10. However, pre-exposure to LPS derived from *Escherichia coli* (LPS_1_) 3 weeks before, significantly attenuated hypothalamic IL-1ra, IL-6 and IL-10 expression. IL-1β expression also tended to be lower. This pattern contrasted with the robust cytokine expression in the spleen of LPS_2_-treated rats previously exposed to LPS_1_, since pre-treatment with endotoxin resulted in a significantly greater response of IL-1β and IL-1ra to LPS_2_. Expression of TNFα and IL-10 also tended to be higher. Pre-treatment with LPS_1_ did not significantly affect the marked increase in corticosterone and adrenaline blood levels induced by LPS_2_. Thus, while endotoxin pre-exposure seemed not to induce a “tolerant” state in the periphery as judged by the immune and endocrine parameters evaluated upon re-stimulation, expression of four of the six cytokines measured was decreased in the hypothalamus. This is the first demonstration that endotoxin priming can differentially affect cytokine expression in the central nervous system and peripheral tissues when a host is confronted with a second, acute, pro-inflammatory stimulus. These results may provide new evidence for the involvement of cytokine pathways in the central nervous system in modulating peripheral inflammation and mediating cognitive and behavioural alterations during inflammatory diseases.

## 1 Introduction

Lipopolysaccharide (LPS), an endotoxin derived from the cell wall of Gram-negative bacteria, is an immunological stressor that stimulates the activity of the hypothalamus–pituitary–adrenal (HPA) axis (Harbuz, 2002) and the sympathetic nervous system (SNS) (Zhang et al., 2000). LPS also induces a rapid inflammatory reaction through the release of pro-inflammatory cytokines, a phenomenon that has been associated with induction or exacerbation of disease in rat models of chronic inflammation (Stimpson et al., 1987; Yoshino et al., 1999). However, in contrast to this acute inflammatory response, we have observed that a single injection of LPS administered 3 weeks prior to the induction of adjuvant arthritis (AA) in rats can confer resistance to inflammation (Harbuz et al., 2002). In parallel to the effects on the HPA axis and SNS, immunological challenge can activate other central pathways such as cytokine networks. Rapid effects of acute injection of LPS on hypothalamic cytokine expression and protein content are well documented (Breder et al., 1994; Laye et al., 1994; Pitossi et al., 1997; Wong et al., 1997; Quan et al., 1998; Castanon et al., 2004; Roche et al., 2006). In addition to this acute stimulation of central cytokine expression, we have recently reported that IL-1β and IL-6 gene expression is increased in the hypothalamus following immunization of rats with arthritogenic type II collagen antigens. Increased expression of these cytokines is maintained for 2–3 weeks, but is no longer detected when symptoms of arthritis are overtly expressed (del Rey et al., 2008). Furthermore, administration of a single dose of IL-1β, a cytokine induced by LPS administration, results in a long lasting (weeks) increased responsiveness of the HPA axis (Schmidt et al., 2001). However, the potential for pre-treatment with LPS to induce long-term changes in SNS activity and in cytokine expression in the hypothalamus has not been studied. The investigation of this possibility is relevant considering that the brain can affect immune processes and mechanisms that underlie cognitive and behavioural alterations during inflammatory diseases.

We have previously observed that the protective effect of prior exposure to LPS on induction of adjuvant-induced arthritis in rats is associated with an alteration in peripheral cytokine secretion (Richards et al., 2006). The main aim of the experiments reported here was to study if prior exposure to LPS affects cytokine gene expression in the hypothalamus and corticosterone and catecholamine output when a host is confronted with a second, acute, pro-inflammatory insult. Furthermore, these studies represent a first attempt to investigate whether the protective effect of LPS on experimentally induced arthritis may also involve changes in brain-borne cytokines. Following the previous protocol (Richards et al., 2006), rats received LPS derived from *Escherichia coli* followed 3 weeks later by a second injection of endotoxin derived from *Salmonella enteritidis*. There were several reasons to choose this combination of LPS from different Gram-negative bacteria as well as the time schedule of exposure. Firstly, we wished to follow an established protocol which demonstrated that, when these two endotoxins were injected 3 weeks apart, the second endotoxin challenge robustly stimulated blood levels of TNFα and IL-10, and also HPA axis activity (Richards et al., 2006). Therefore there was no evidence that an endotoxin from an alternative source was acting as a homotypic stressor, thus inducing HPA axis habituation (Dallman et al., 2002). Secondly, we wanted to use a second stimulus that also acts via Toll 4-like receptors to avoid the possibility that stimulation of other Toll-like receptors would induce the production of cytokines different from those triggered by the first Toll 4 agonist (Ghosh et al., 2006). Finally, while the effects of LPS on the HPA axis and the SNS are rather well known and established, there is still controversy regarding endocrine responses induced by stimuli of other Toll-like receptors (Myers et al., 2001).

Thus, using this established model, expression of the same cytokines was evaluated in the hypothalamus and in the spleen to compare central and peripheral responses to LPS pre-treatment. Peripheral catecholamine output and corticosterone blood levels were also determined in parallel.

## 2 Materials and methods

### 2.1 Animals

Adult male Wistar rats (200–225 g; Bantin & Kingman, UK) were housed under standard conditions of temperature and humidity with 12 h:12 h light/dark cycle, lights on at 07.00 h. Animals were fed laboratory chow and water *ad libitum* throughout experiments. The care and use of the animals was performed in accordance with the Animals (Scientific Procedures) Act UK 1986.

### 2.2 Experimental procedures

Groups of rats (*n* = 8) received endotoxin-free saline (SAL; 0.5 ml) or lipopolysaccharide (LPS_1_; Sigma, UK, serotype *E. coli* 055:B5; 1 mg/kg body weight in 0.5 ml of saline) injected intraperitoneally between 8:00 and 8:30 a.m. and returned to their home cages. This dose of LPS of the 055:B5 serotype is sufficient to stimulate the HPA axis with no apparent effects on animal behaviour, e.g., locomotion, fur appearance, exploratory behaviour (Conde et al., 1999). Twenty-one days later, both groups of animals were injected i.p. with LPS derived from *S. enteritidis* (LPS_2_; 1 mg/kg body weight, 100K4088, Sigma, UK) in 0.5 ml of saline between 8:00 and 8:30 a.m. (SAL + LPS_2_; and LPS_1_ + LPS_2_). The dose of Salmonella enteritidis LPS that was used in the present study has no observable behavioural effects. A control group of rats were injected with endotoxin-free saline, and they received a second injection of saline on day 21 (SAL + SAL). We did not include the control group LPS_1_ + SAL because we have previously observed that blood cytokine and hormone levels in this control group after 21 days were no different to those in the group SAL + SAL (Richards et al., 2006), i.e., were all basal. Similarly, gene expression of several cytokines (IL-1β, IL-6, TNFα and IFN-γ) in several brain regions and in the spleen following administration of LPS were decreasing or had returned to basal after 6 h (Pitossi et al., 1997). Although these studies were performed in mice, we considered it to be extremely unlikely that cytokine gene expression in the hypothalamus or spleen of rats would still be elevated 21 days following LPS.

Following the second injection, rats were returned to their home cages and sacrificed by decapitation 4 h later. Trunk blood was collected in heparinised tubes, centrifuged, and the plasma was aliquoted and stored at −80 °C until used for corticosterone and catecholamine measurements. Brains and spleens were removed and immediately frozen on dry ice. The hypothalamus (optic chiasm laterally, mammillary bodies posteriorly, fornix laterally and top of the third ventricle) was dissected from the rest of the brain and tissues were stored at −80 °C for cytokine mRNA analysis.

### 2.3 Corticosterone and catecholamine measurements

Corticosterone plasma concentrations were determined by RIA, as previously described (Besedovsky et al., 1991). Noradenaline (NA), adrenaline (A) and dopamine (DA) concentrations were determined by HPLC as previously described (del Rey et al., 2008). Briefly, plasma samples were treated with 0.4 M perchloric acid and underwent a purification step with alumina adsorption prior to HPLC determination. Aliquots of the supernatant were injected into an HPLC system with serially connected electrochemical and fluorescence detection. Peaks were quantified by peak height evaluation with an evaluation software (Chromeleon Version 6.01, Dione, USA).

### 2.4 RNA extraction and RT-PCR

These procedures were performed as previously described (Apkarian et al., 2006). Briefly, total RNA extraction was performed using TRIzol Reagent (Invitrogen Life Technologies) according to a standard protocol (Chomczynski and Sacchi, 1987). The RNA was treated with 2U DNaseI (Epicentre technologies) in 10× Buffer Y+/Tango (MBI Fermentas) followed by purification using RNeasy Mini Spin Columns (Qiagen) according to the manufacturer’s instruction and eluted in 30 μl RNase free water. Reverse transcription (RT) was performed from 1 μg total RNA using 40U MMLV reverse transcriptase (Invitrogen Life Technologies) and 0.5 mg/ml oligop(dT) 12-18-primer (Amersham Biosciences) in a total volume of 20 μl. RT was performed at 42 °C for 60 min and 70 °C for 15 min. PCR was performed in a volume of 25 μl with the ABI PRISM 7700 Sequence Detection System (PE Applied Biosystems) using optical reaction tubes. A master mix was prepared containing 12.5 μl 2× PCR buffer (100 mM KCl, 20 mM Tris–HCl pH 8.3, 0.02 mM EDTA, 0.1% gelatin, 0.02% Tween-20), nucleotides dATP, dCTP, dGTP (200 μM each), 400 μM dUTP, 1.0 μl 25 mM MgCl2, 0.625U AmpliTaqGold (PE Applied Biosystems), 0.25U Uracil- DNA-Glycosylase (New England Biolabs), 200 nM of each primer, 100 nM of the corresponding probe, and Rox dye in a final concentration of 300 nM (TIB MOLBIOL). The master mix (21 μl) were added to each well of 96 well-plates followed by addition of 4 μl cDNA. All PCR reactions were performed two to four times in duplicates using the following conditions: initial 50 °C for 2 min and 95 °C for 10 min, followed by 40 cycles at 95 °C for 15 s and 60 °C for 1 min.

### 2.5 Relative quantitation of PCR products

Primer and fluorogenic probes were designed using the automated primer analysis software, Primer Express (PE Applied Biosystems). Primer and probes were chosen to bind in different exons or to span exon junctions to prevent amplification of genomic DNA. The forward and reverse primers, and internal probe sequences for each cytokine and the house keeping genes are given in Table 1 (for rpL32 (Wang et al., 2000); for TNFα, (Fink et al., 1998); for IL-10 (Overbergh et al., 2003). The comparative CT method, previously described (Livak et al., 1995) was used to calculate relative gene expression data since we have determined in preliminary experiments that the amplification efficiencies of the target genes and the reference genes (rpL32 and GAPDH) are approximately the same. Thus, cytokine mRNA levels were normalized to the rpL32 (hypothalamus) and GAPDH (spleen) mRNA levels in each sample. Unless otherwise indicated, the value of saline-injected animals was arbitrarily set at 1.0, and the results of the groups (SAL + LPS_2_) and (LPS_1_ + LPS_2_) expressed as fold-change. To compare hypothalamic and splenic cytokine expression following re-exposure to LPS (LPS_1_ + LPS_2_ group) with the expression induced by a single injection of endotoxin, the value of (SAL + LPS_2_)-injected animals was arbitrarily set at 1.0, and the results of the (LPS_1_ + LPS_2_) group expressed as fold-change in each corresponding tissue.

### 2.6 Statistical analysis

Results are expressed as mean ± SEM. Data were analyzed using one-way ANOVA followed by Fisher’s test for multiple comparisons. Differences were considered significant when *p* was <0.05.

## 3 Results

### 3.1 Corticosterone and catecholamine blood concentrations

**Fig. 1:**
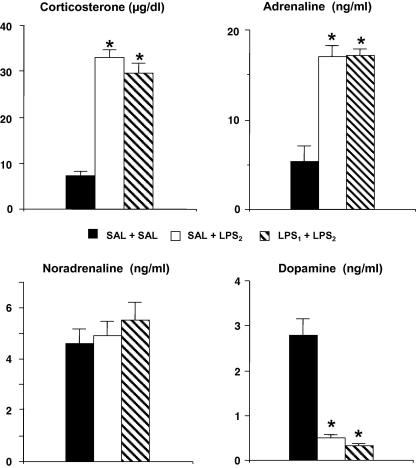
Corticosterone and catecholamine levels in plasma following one or two injections of LPS from different sources. Groups of rats received endotoxin-free saline (SAL) or lipopolysaccharide from *Escherichia coli* (LPS_1_) injected intraperitoneally. Twenty-one days later, both groups of animals were injected i.p. with LPS derived from *Salmonella enteritidis* (LPS_2_) (SAL + LPS_2_ and LPS_1_ + LPS_2_). A third group of rats injected with endotoxin-free saline 21 days before, received a second injection of saline (SAL + SAL). Animals were sacrificed 4 h after the second injection. Corticosterone and catecholamine levels were determined in plasma. Bars indicate the mean ± SEM of determinations performed in 7–8 animals per group. ∗*p* < 0.05 vs. SAL + SAL.

Corticosterone plasma concentrations were increased in response to the injection of LPS_2_ in both groups of rats that received LPS (SAL + LPS_2_ and LPS_1_ + LPS_2_) compared to the SAL + SAL controls, but no significant difference was observed between them (Fig. 1). NA plasma concentrations were unaffected by LPS, but a significant increase in A, and decrease in DA, concentrations was observed in response to LPS_2_ (Fig. 1) compared to the SAL + SAL controls. There were no significant differences between the SAL + LPS_2_ and LPS_1_ + LPS_2_ groups for any catecholamine measured.

### 3.2 Cytokine gene expression in the hypothalamus

**Fig. 2:**
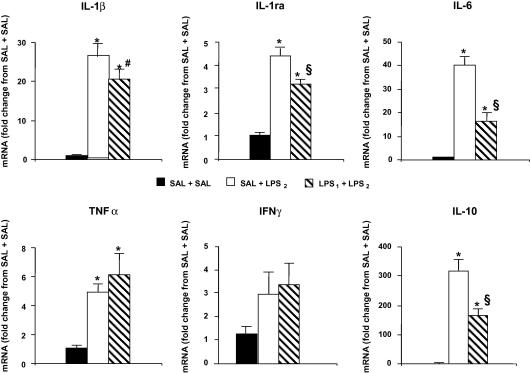
Cytokine gene expression in the hypothalamus following one or two injections of LPS from different sources. The hypothalami of the rats used for the determinations shown in Fig. 1 were dissected from brains frozen on dry ice immediately after decapitation, and stored at −80 °C until used for cytokine mRNA determinations by real time RT-PCR. Bars indicate the mean ± SEM of the results expressed as fold-change from the expression in the SAL + SAL control group. ∗*p* < 0.05 vs. SAL + SAL; §*p* < 0.05 vs. SAL + LPS_2_; #*p* = 0.117 vs. SAL + LPS_2_.

Significant increases were observed in mRNA for all cytokines measured in the SAL + LPS_2_ and LPS_1_ + LPS_2_ groups when compared with SAL + SAL controls, with the exception of IFNγ, for which the difference did not reach statistical significance (Fig. 2). Pre-treatment with LPS_1_ resulted in significantly less IL-1ra, IL-6 and IL-10 gene expression in the LPS_1_ + LPS_2_ group compared to the SAL + LPS_2_ group. A decrease in IL-1β mRNA, and increase in TNFα mRNA, were observed in the LPS_1_ + LPS_2_ group compared to the SAL + LPS_2_ group, but the differences did not reach statistical significance.

### 3.3 Cytokine gene expression in the spleen

**Fig. 3:**
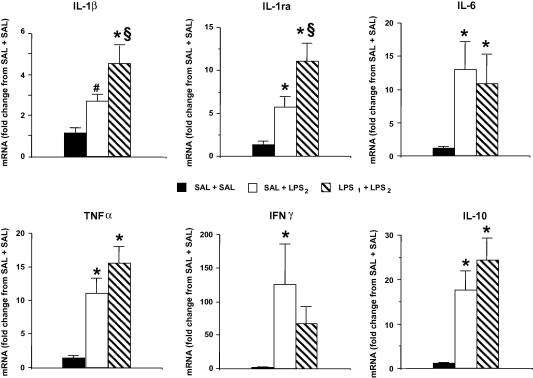
Cytokine gene expression in the spleen following one or two injections of LPS from different sources. The spleen of the rats used for the determinations shown in Figs. 1 and 2 was dissected immediately after decapitation, frozen and stored at −80 °C until used for cytokine mRNA determinations by real time RT-PCR. Bars indicate the mean ± SEM of the results expressed as fold-change from the expression in the SAL + SAL control group. ∗*p* < 0.05 vs. SAL + SAL; §*p* < 0.05 vs. SAL + LPS_2_; #*p* = 0.055 vs. SAL + SAL.

Injection of LPS_2_ alone resulted in significant increases in IL-1ra, TNFα, IFNγ, IL-6 and IL-10 mRNA in the spleen when compared to the SAL + SAL controls (Fig. 3). The increase in IL-1β mRNA levels induced by LPS_2_ was nearly statistically significant (*p* = 0.055 vs. Sal + Sal). With the only exception of IFNγ, exposure to LPS_1_ 3 weeks prior to LPS_2_ administration resulted either in a similar (IL-6, TNFα and IL-10) or even increased (IL-1β and IL-1ra) cytokine gene expression in the spleen when compared to those of rats that received LPS_2_ only.

### 3.4 Re-exposure to LPS differentially affects cytokine gene expression in the hypothalamus and in the spleen

**Fig. 4:**
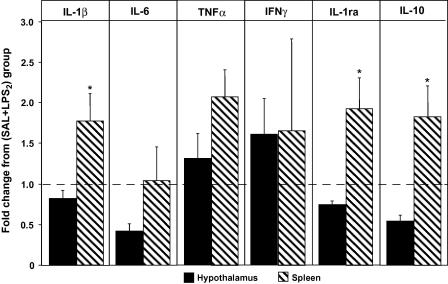
Re-exposure to endotoxin results in a different pattern of cytokine gene expression in the hypothalamus and in the spleen. For a better appreciation of the different effects that re-exposure to endotoxin has on cytokine gene expression in the hypothalamus and spleen, the results obtained for each tissue have been normalized to SAL + LPS_2_ and expressed as fold-change from this group. Thus, values <1 indicate that re-exposure to endotoxin (LPS_1_ + LPS_2_) results in less expression (respectively, values >1 indicate more expression) of a given cytokine in a given tissue than one single exposure (SAL + LPS_2_). As can be appreciated, re-exposure to endotoxin results in less IL-1β, IL-6, IL-1ra, and IL-10 gene expression in the hypothalamus, while IL-1, IL-1ra and IL-10 expression in the spleen is increased. Bars indicate the mean ± SEM. ∗*p* < 0.05 vs. expression in hypothalamus.

Differential effects of a second LPS challenge on cytokine expression in the hypothalamus and spleen are summarized in Fig. 4. To compare the impact that prior exposure to LPS (LPS_1_) has on the capacity of the hypothalamus and the spleen to express pro- and anti-inflammatory cytokines when a host is confronted with a second pro-inflammatory stimulus (LPS_1_ + LPS_2_), the results of the group (SAL + LPS_2_) in both tissues were set at 1.0 and expressed as fold-change (Fig. 4). Thus, values lower than 1 indicate that re-exposure to LPS results in less gene expression, and values higher than 1 in more expression, of a given cytokine in a given tissue as compared to one single exposure. The results show that re-exposure to LPS differentially affects the expression of most of the cytokines evaluated in the hypothalamus and in the spleen. While re-exposure to LPS resulted in more IL-1β, TNFα, IFNγ, IL-1ra, and IL-10 gene expression in the spleen, the expression of IL-1, IL-1ra and IL-10 was decreased in the hypothalamus. Comparatively, LPS re-exposure also resulted in less IL-6 and TNFα expression in the hypothalamus than in the spleen, but the differences did not reach statistical significance. IFNγ expression showed a larger variance between animals than the other cytokines and it was similar in both organs. The maximal differences were observed in the two anti-inflammatory cytokines, IL-1ra and IL-10. In both cases, the spleen showed more than two-fold increased expression of mRNA transcripts than the hypothalamus.

## 4 Discussion

This is the first report that prior exposure to LPS can alter gene expression of cytokines in the hypothalamus of rats when they are challenged with a second injection of LPS derived from another Gram-negative bacteria. A further novel observation is that, in contrast to the general decrease in hypothalamic cytokine expression in LPS-pre-treated rats, an increase in most cytokines measured was observed in the spleen.

In response to a single injection of LPS, we observed increased hypothalamic expression of the pro-inflammatory cytokines IL-1β, IL-6, and TNFα, which is consistent with the acute cytokine response to LPS previously reported (Breder et al., 1994; Laye et al., 1994; Pitossi et al., 1997; Wong et al., 1997; Quan et al., 1998; Castanon et al., 2004). The expression of the anti-inflammatory cytokines IL-10 and IL-1ra was also increased. The dose of LPS that we employed was less than that required to induce sickness behaviour in rats (Conde et al., 1999). Furthermore, we have previously reported that even double the dose used in this study does not disrupt the blood–brain barrier in rats (Bickel et al., 1998). Therefore, it is not likely that the cytokine responses that we observed following a single injection of LPS were the consequence of an inflammatory response to tissue damage caused by disruption of the blood–brain barrier or to sickness behaviour, which can be induced by higher doses of LPS or similar doses of a different serotype (Castanon et al., 2001).Exposure of the LPS-pre-treated rats to a second injection of LPS also resulted in increased expression of IL-1β, IL-6, TNFα, IL-1ra and IL-10 in the hypothalamus when compared to rats that received the vehicle alone. However, although LPS-pre-treatment did not significantly affect hypothalamic TNFα and IFNγ expression, it significantly decreased the expression of IL-6, IL-10 and IL-1ra when compared to non-pre-treated rats. IL-1β gene expression also tended to decrease, but no statistical significance was reached (*p* = 0.117). In contrast to the hypothalamus, re-exposure to LPS resulted in increased splenic IL-1β and IL-1ra expression when compared to a single endotoxin injection, while the increase in IL-6, TNFα and IL-10 was comparable whether rats had been pre-treated or not.Using the same model as in this study, we have previously reported that re-exposure to LPS results in decreased blood levels of IFNγ and IL-6, without affecting TNFα and IL-10 concentrations (Richards et al., 2006). This pattern of circulating cytokine levels is essentially consistent with an anti-inflammatory milieu that might explain the protective effect of the endotoxin on the adjuvant arthritis model (Harbuz et al., 2002). On one hand, over-expression of IL-1ra, a tendency to decreased IFNγ expression and no changes in IL-10 expression in the spleen of rats re-exposed to LPS is in line with our previous report. On the other hand, a second inflammatory insult increased the gene expression of the pro-inflammatory cytokine IL-1, while it did not significantly affect the expression of IL-6 and TNFα. Tentative explanations for the apparent discrepancy in IL-6, which was decreased in the circulation but no changes were observed in splenic gene expression, could be that a second endotoxin challenge results in inhibition of cytokine production at translational levels or in its secretion by immune cells. In addition, a reduction in the concentration of pro-inflammatory cytokines in the circulation may be the result of effects in all immune cell compartments while the studies reported here were restricted to the evaluation of splenic cytokine expression. In certain models of endotoxin tolerance, for example, it has been shown that splenic mRNA IL-1 levels are increased following LPS injection, but that the spleen is not essential for the increase in serum IL-1 concentration (Zuckerman et al., 1991). In any case, at least at gene level, our data demonstrate that the hypothalamus and the spleen respond with a different pattern of cytokine expression to a second pro-inflammatory insult. As mentioned, the maximal relative differences between these two tissues were observed in the two anti-inflammatory cytokines, IL-1ra and IL-10.Very little has been published on the impact of previous exposure to LPS on cytokine gene expression in the brain following re-exposure to LPS. Previous reports are largely related to the phenomenon of “endotoxin tolerance”, a term which is frequently used to define a condition in which re-exposure to a second, much higher, dose of endotoxin after a short time is associated with blunted peripheral immune and HPA axis responses (Roth et al., 1994; Faggioni et al., 1995; Hadid et al., 1995, 1996; Takemura et al., 1997; Grinevich et al., 2001; Beishuizen and Thijs, 2003; Chen et al., 2005). Mice pre-treated with LPS showed attenuated brain and serum TNFα responses to a second injection of LPS 4 days later (Faggioni et al., 1995). Other authors reported that central TNFα and IL-1β expression were similar in response to a high dose of LPS in pre-treated and non-pre-treated rats over a 9 day experimental period while IL-6 mRNA was decreased in the pre-treated group (Chen et al., 2005). In both these studies, cytokine mRNA was measured in whole brain extracts. The effects of endotoxin tolerance on cytokine release by immune cells and HPA axis activity have been reported up to 28 days following initial exposure to endotoxin (Roth et al., 1994; Hadid et al., 1995, 1996; Takemura et al., 1997; Grinevich et al., 2001). In accord with our previous results (Richards et al., 2006), the data reported here also show that in the model we have used, prior exposure to LPS does not seem to result in “peripheral tolerance” when animals are exposed again to an endotoxin from a different bacterial source. Probably the strongest argument against the possibility that our findings reflect the development of endotoxin tolerance is the fact that priming with LPS 21 days prior to a second LPS challenge resulted in similar increases of IL-6, TNFα and IL-10 gene expression in the spleen, and corticosterone and adrenaline levels in blood, and even increased splenic IL-1β and IL-1ra gene expression, compared to a single LPS injection. If there is any “tolerance” induction in the model we have used, this is only observed at hypothalamic level, with decreased IL-1β, IL-1ra, IL-6 and IL-10 gene expression.

In addition to activating the HPA axis, a single dose of LPS also stimulates the SNS (Conde et al., 1999; Zhang et al., 2000). We observed similar effects of LPS on plasma adrenaline, while no effect was observed on noradrenaline. The decrease in dopamine levels observed in parallel might reflect an increase in adrenaline turnover in the adrenal gland. Peripheral catecholamines have been shown to modulate the effects of LPS on peripheral immune functions (Delrue-Perollet et al., 1995). Therefore, the fact that the response of the SNS is maintained after re-exposure to LPS would be an additive component to the anti-inflammatory cytokine milieu that we have previously detected. Taken together, the preserved neuro-endocrine response (increase of adrenaline and glucocorticoid blood levels) to a second pro-inflammatory insult may contribute to prevent the induction of inflammatory disease in rats pre-exposed to LPS (Shanks et al., 2000; Harbuz et al., 2002) or IL-1 (Huitinga et al., 2000).A wide range of functions have been ascribed to cytokines within the brain (Besedovsky and del Rey, 2008). In addition to the evidence that peripherally immune-derived cytokines can trigger neuro-endocrine responses during infection and inflammation, several non-immune functions of cytokines such as TNFα, IL-1β and IL-6 synthesised within the CNS in the absence of disease have been described. For example, it is well documented that expression of cytokines within a physiologic range is relevant for learning, memory and behaviour, neuroprotection and the control of neuro-endocrine functions (Besedovsky and del Rey, 2007; Gosselin and Rivest, 2007) and it may be inappropriate to categorize these CNS cytokines as pro- or anti-inflammatory in the classic immune sense, particularly in the lower concentrations in which they are found in the “healthy” CNS compared to immune tissues. On the other hand, over-expression of brain-borne cytokines can mediate alterations in cognitive and behavioural mechanisms that occur during peripheral autoimmune/inflammatory pathologies. Our findings that sequential pro-inflammatory insults result in decreased expression of IL-1ra and IL-10, cytokines that can modulate the effect of inflammatory mediators in the hypothalamus, may further contribute to aggravate the alterations referred to. For example, the effects of IL-1β on serotonergic pathways that are involved in depressive symptoms are well known (for review see, Besedovsky and del Rey, 2008). Differential central cytokine responses to a single or repeated LPS stressor may also confer potential for a paracrine influence of cytokines within the hypothalamus, for example, on CRH and/or AVP expression for fine-tuning of HPA axis activity, which can in turn affect the immune system through catecholamine and glucocorticoid release*.* In fact central administration of low doses of IL-1, which are not effective in the periphery, results in immunosuppression that is at least partially dependent on an increased secretion of adrenal hormones (Sundar et al., 1989).

One possibility to interpret the results reported here is that the decrease in anti-inflammatory cytokines in the brain is not the cause but the consequence of a blunted peripheral inflammatory response. Indeed, we and others have previously shown that the brain, particularly the hypothalamus, can sense ongoing immune responses (Besedovsky et al., 1983; Furukawa et al., 2004; for review see Besedovsky and del Rey, 1996) and, when the immune response is of sufficient magnitude, a neuro-endocrine immuno-regulatory “answer” is elicited. We have also hypothesized that the pattern of central expression of cytokines is a way to codify and register what type of peripheral immune response is in course. This way of central representation of immune system activity would constitute a kind of “immune homunculus” in the CNS (Besedovsky and del Rey, 2001). The fact that, 3 weeks after a first stimulus, a second inflammatory challenge with an LPS from a different bacterial origin elicits a change in the pattern of expression of cytokines in the hypothalamus, suggests that a kind of “memory” is stored in the brain. We speculate that the reduction in cytokine expression in the hypothalamus reflects the way by which the brain” remembers” a previous inflammatory insult.

While, from these data, we cannot draw functional conclusions about effects of central cytokines, the data justify further studies of hypothalamic cytokine expression in animal models of “peripheral” inflammatory pathologies. In particular, the influence of prior exposure to pro-inflammatory agents on central cytokine expression may provide insights into the relevance of effects of hypothalamic cytokines on CNS and neuro-endocrine functions during remission/relapse episodes of peripheral inflammatory chronic diseases and on the neuropsychiatric components of these pathologies.

## Figures and Tables

**Table 1:** Primer and probe sequences used to quantify cytokine and reference gene expression.

Accession	Name	Forward Primer	Probe	Reverse Primer
X06483	rpL32	TGTCCTCTAAGAACCGAAAAGCC	TCGTAGAAAGAGCAGCACAGCTGGCC	CGTTGGGATTGGTGACTCTGA
X02231	GAPDH	ACGGGAAACCCATCACCAT	TTCCAGGAGCGAGATCCCGTCAAG	CCAGCATCACCCCATTTGA
E05490	IL-1ß	ACCCAAGCACCTTCTTTTCCTT	TCTTTGAAGAAGAGCCCGTCCTCTGTGACT	TGCAGCTGTCTAATGGGAACAT
X66539	TNFα	GGTGATCGGTCCCAACAAGGA	TGGCCCAGACCCTCACACTCAGATCA	CACGCTGGCTCAGCCACTC
NM_012589	IL-6	GACAGTGCATCATCGCTGTTCATA	CAGAATTGCCATTGCACAACTCTTTTCTCATTT	AGTCGGAGGCTTAATTACATATGTTC
AF010466	IFNγ	GCTATGGAAGGAAAGAGCCTCC	ATATCTGGAGGAACTGGCAAAAGGACGGT	GATGGCCTGGTTGTCTTTCAA
M63101	IL-1ra	CTCTCCTTCTCATCCTTCTGTTTC	AAGATGCAAGCCTTCAGAATCTGGGATACT	AGCAATGAGCTGGTTGTTCCTC
X60675	IL-10	GGTTGCCAAGCCTTGTCAGAA	TGCGACGCTGTCATCGATTTCTCCC	GCTCCACTGCCTTGCTTTTATT
